# Patient Characteristics and Outcomes of Endoscopically Placed Dedicated Transgastric Jejunal Tubes Compared to Percutaneous Endoscopic Gastrostomy With Jejunal Extension Tubes

**DOI:** 10.1002/deo2.70213

**Published:** 2025-09-26

**Authors:** Laura D. Kek, David H. Bruining, Navtej S. Buttar, Hadi Abou Zeid, Andrew C. Storm, William S. Harmsen, Louis M. Wong Kee Song, Nayantara Coelho‐Prabhu

**Affiliations:** ^1^ Division of Internal Medicine Mayo Clinic Rochester Minnesota USA; ^2^ Division of Gastroenterology and Hepatology Mayo Clinic Rochester Minnesota USA

**Keywords:** enteral nutrition, gastrostomy, jejunostomy, risk factors, treatment outcome

## Abstract

**Objectives:**

Patients requiring long‐term enteral nutrition or continuous infusion of carbidopa/levodopa can benefit from jejunostomy tube placement. Recently, directly placed percutaneous transgastric jejunal tubes (TGJs) have been used instead of gastrostomy tubes with jejunal extensions (PEG‐Js) for enteral access. We aim to compare patient characteristics and outcomes after placement of TGJs placed via the introducer technique compared to PEG‐Js.

**Methods:**

We performed a retrospective study of 141 patients (TGJ = 58, PEG‐J = 83) assessed at Mayo Clinic between 2010 and 2024. Patients were identified using a prospectively maintained procedure data registry. Demographic data, patient characteristics, procedural indications, complications, and first‐replacement date were gathered. Statistical analysis included the Wilcoxon rank sum test, chi‐square test, Fisher's exact test, and Kaplan‐Meier estimates. Patients receiving carbidopa/levodopa were excluded from complications analysis due to the carbidopa/levodopa tube's proprietary structure. A *p*‐value of <0.05 was set as a threshold for significance.

**Results:**

Our results demonstrated no difference in cumulative incidence of complications within 1 year for TGJs and PEG‐Js, *p*‐value 0.48. Regarding time to first replacement, treating death as a competing risk factor, there was no statistically significant difference in cumulative incidence of replacement within 1 year for TGJs and PEG‐Js, *p*‐value 0.389.

**Conclusions:**

Our study demonstrates that both direct TGJs and PEG‐Js are safe options for long‐term jejunal feeding. More studies are needed to compare endoscopic to radiologically placed percutaneous transgastric jejunal feeding tubes.

## 1 Introduction

Patients who require long‐term (greater than 4 weeks) post‐pyloric nutrition or continuous infusion of certain medications, such as carbidopa/levodopa, undergo percutaneous tube placement. In some patients, conditions such as gastric outlet obstruction due to tumor, stricture, or pancreatic inflammation, delayed gastric emptying or gastroparesis, and recurrent aspiration pneumonia, preclude the use of gastric feeding and necessitate feeding into the jejunum. The type of tube placed and the method by which it is placed are determined on a case‐by‐case basis, keeping the patient's indication and clinical context in mind.

**FIGURE 1:**
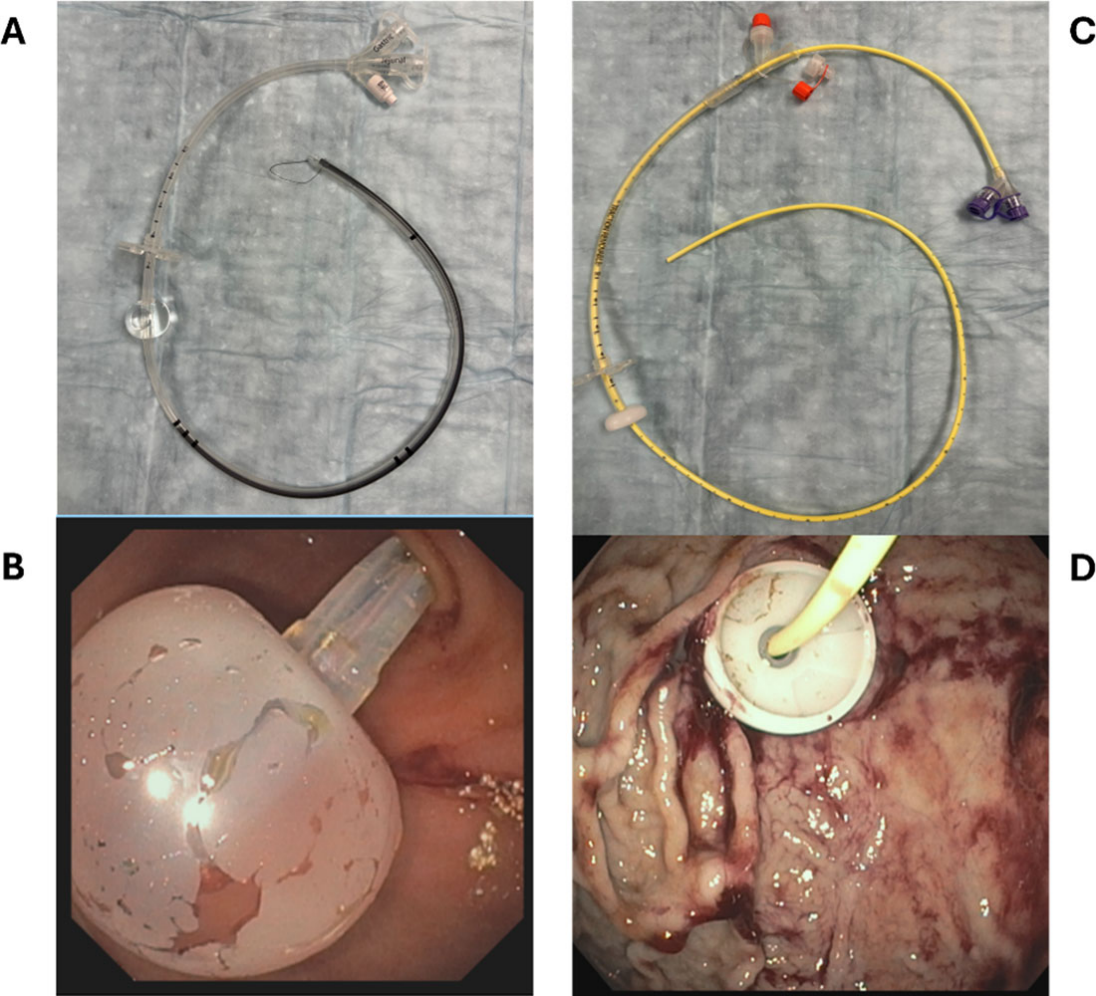
Transgastric jejunal (TGJ) and percutaneous endoscopic gastrostomy with jejunal extension (PEG‐J) tubes. 2A and 2B demonstrate the TGJ tube outside (2A) and inside (2B) of the body. 2C and 2D demonstrate the PEG‐J tube outside (2C) and inside (2D) of the body.

The most common forms of jejunal access include direct percutaneous endoscopic jejunostomy (DPEJ) tubes, percutaneous endoscopic gastrostomy with jejunal extension (PEG‐J) tubes, and dedicated transgastric jejunal (TGJ) tubes. TGJs do not involve a separate jejunal extension tube, but rather a single‐piece tube which enters percutaneously through the stomach and is advanced into the jejunum under endoscopic or fluoroscopic guidance by Gastroenterology or Interventional Radiology (IR) proceduralists (Figure 1) [1]. Previously, TGJ tubes were sometimes placed as replacements for PEG‐J tubes after an interval of at least 4 weeks [2]. A relatively newer technique is the Introducer technique, which allows for the initial placement of a single‐piece TGJ tube. This endoscopic procedure involves initial gastropexy of the stomach to the anterior abdominal wall with T‐fasteners. Then, a direct percutaneous dilating catheter and sheath are passed over a guidewire until the outermost sheath is introduced into the stomach. Thereafter, the guidewire is either dragged down into the jejunum with an orally passed endoscope or an ultrathin endoscope is passed percutaneously through the sheath directly into the jejunum for placement of the guidewire in the jejunum. Finally, the single‐piece TGJ tube (≥18 Fr) is passed over the wire into the jejunum, the internal balloon bumper of the TGJ tube is inflated in the stomach, and the outer sheath is peeled away [3]. For PEG‐J placement, a standard pull‐through technique is utilized to place a PEG tube with an internal disk bumper, usually larger than 18 Fr diameter. Through this PEG tube, a 9 Fr jejunal extension tube is introduced into the stomach and dragged into the jejunum either directly or over a guidewire using an orally passed endoscope. At times, this jejunal extension tube is anchored to the jejunal wall by endoscopic clips to prevent its retraction into the stomach. Complications of both PEG‐J and TGJ tubes include tube clogging, migration, volvulus, and infection [1, 2].

While the complications and indications of gastric versus jejunal feeding are well‐documented, the outcomes of the endoscopic Introducer technique (TGJ) compared to endoscopic PEG‐J tube placement have not been reported. Herein, we assess the patient characteristics and outcomes after initial placement of TGJs placed via the Introducer technique compared to PEG‐Js.

## 2 Methods

We performed a retrospective study of adult patients who underwent initial Introducer technique (TGJ) or Pull technique (PEG‐J) placement at Mayo Clinic between 2010 and 2024. Patients were identified using a prospectively maintained procedure registry. We extracted demographic data, patient characteristics, procedural indications, intra‐procedural complications, post‐procedural complications, and first replacement date. Indications were broadly categorized into chronic motility disorders, gastroduodenal obstruction, acute hospitalization, carbidopa/levodopa administration, pancreas‐associated conditions, intolerance to oral diet, and esophageal‐related disorders. Post‐procedural complications of interest were tube dislodgement, flipping, and clogging. These events were gathered from a chart review of procedural documentation. Patients receiving carbidopa/levodopa were excluded from complications analysis due to the carbidopa/levodopa tube's proprietary structure. Statistical analysis included the Wilcoxon rank sum test, chi‐square test, Fisher's exact test, and Kaplan‐Meier estimates. A *p*‐value of <0.05 was set as a threshold for significance.

## 3 Results

We identified 141 patients (TGJ = 58, PEG‐J = 83) with initial placement. Table 1 describes the patient characteristics. Mean age at TGJ and PEG‐J placement was 60.4 ± 16.2 years and 62.3±15.0 years, respectively. Mean pre‐procedural BMI for patients receiving TGJs and PEG‐Js was 24.5 ± 5.8 and 23.8 ± 5.5 kg/m^2^, respectively. Diabetes mellitus was a concomitant diagnosis in 24.8% of patients. In the PEG‐J cohort, 11.0% had a history of Billroth II procedure.

**TABLE 1:** Demographics and patient characteristics.

	PEG‐J (*N* = 83)	TGJ (*N* = 58)	Total (*N* = 141)	*p*‐Value
**Age at Procedure**				0.6690^a^
Mean (SD)	62.3 (15.0)	60.4 (16.2)	61.5 (15.5)	
				
**Sex**				0.3473^b^
Female	41 (49.4%)	24 (41.4%)	65 (46.1%)	
Male	42 (50.6%)	34 (58.6%)	76 (53.9%)	
				
**BMI**				0.5269^a^
Mean (SD)	23.8 (5.5)	24.5 (5.8)	24.1 (5.6)	
				
**DM**				0.0682^b^
No	67 (80.7%)	39 (67.2%)	106 (75.2%)	
Yes	16 (19.3%)	19 (32.8%)	35 (24.8%)	
				
**Prior Surgery**				0.3451^c^
Missing	1	0	1	
No Surgery	68 (82.9%)	54 (93.1%)	122 (87.1%)	
Billroth II	9 (11.0%)	2 (3.4%)	11 (7.9%)	
Roux‐en‐y	3 (3.7%)	2 (3.4%)	5 (3.6%)	
Esophageal Resection	1 (1.2%)	0 (0.0%)	1 (0.7%)	
Gastric Resection	1 (1.2%)	0 (0.0%)	1 (0.7%)	

^a^ Wilcoxon rank sum test.

^b^ Chi‐square test.

^c^ Fisher's exact test.

Regarding indications, in the TGJ group, 10 were placed due to chronic motility disorders, 13 were placed due to gastroduodenal obstructions, seven were placed due to acute hospitalizations, none were placed for carbidopa/levodopa administration or pancreas‐associated conditions, nine were placed due to intolerance to oral diet, and 19 were placed due to esophageal‐related disorders. In the PEG‐J group, 12 were placed due to chronic motility, 25 were placed due to gastroduodenal obstructions, five were placed due to acute hospitalizations, 12 were placed for carbidopa/levodopa administration, three were placed for pancreas‐associated conditions, 13 were placed due to intolerance to oral diet, and 13 were placed due to esophageal‐related disorders. TGJs were placed more often for esophageal conditions (32.8%) compared to PEG‐Js for gastroduodenal obstructions (33.7%) (*p* = 0.0034). 36.9% of all patients had a malignancy‐associated indication for their feeding tube placement; of those, 67.3% received a PEG‐J compared to 32.7% of patients who received a TGJ tube (*p* = 0.1194). Eleven jejunal extension tubes underwent clip anchoring in the proximal jejunum, and none of the TGJ tubes were anchored by clips.

The tube size of the various devices was also collected. The TGJ cohort had 39 patients with size 18 Fr tubes placed and 19 patients with size 22 Fr tubes placed. The PEG‐J cohort had 44 patients with 20 Fr outer tubes, 30 patients with 24 Fr outer tubes, and nine patients with 15 Fr outer tubes. The jejunal extensions in the PEG‐J tubes had various sizes, including 8.5, 9, 10, 12, and 14 Fr.

Additionally, the procedural length was compared, as this may be extrapolated to interpret the possible difficulty of the respective procedures. The average procedural time was 44.05 min for TGJs and 47.21 min for PEG‐J placements.

**FIGURE 2:**
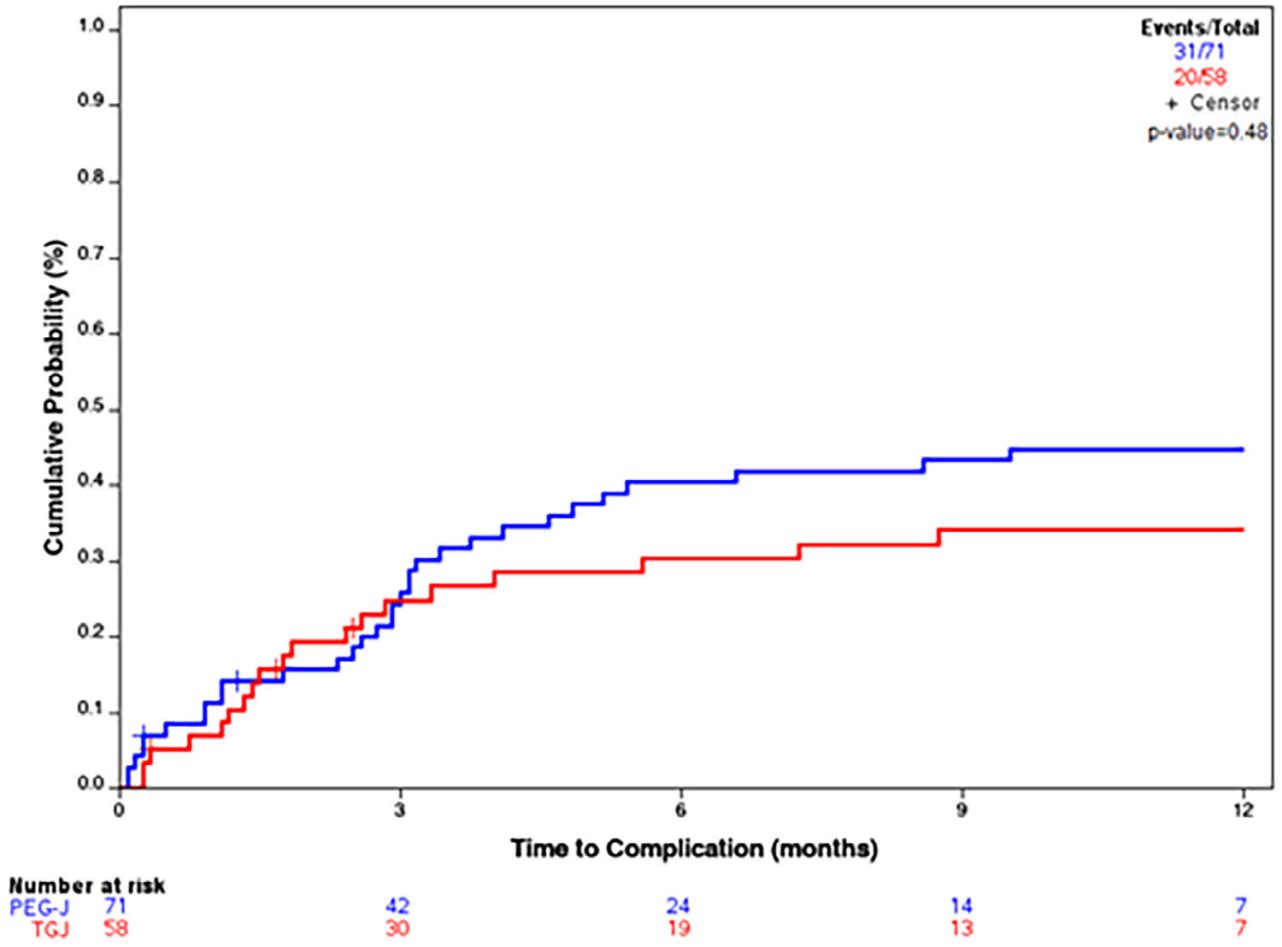
Cumulative incidence of complications within 1 year of percutaneous endoscopic gastrostomy with jejunal extension (PEG‐J) compared to transgastric jejunal (TGJ) tubes. *p*‐Value 0.48.

Regarding intra‐procedural complications, there were two instances of iatrogenic gastric mucosal trauma in the PEG‐J cohort. These were not clinically significant events and did not require further intervention. There was no significant difference in cumulative incidence of post‐procedural complications of tube dislodgement, flipping, and clogging within 1 year of initial placement for TGJs and PEG‐Js (*p* = 0.99) (Figure 2). The hazard ratio (HR) for complications in the PEG‐J group compared to the TGJ group was not significant (HR = 1.295, *p* = 0.3762). Other post‐procedural complications that were collected included bleeding, perforation, site infection, buried bumper, and death. In the TGJ cohort, there were 3 instances of bleeding and no perforations, site infections, buried bumpers, or death. In the PEG‐J cohort, there were 2 instances of buried bumpers, and no bleeding, perforations, site infections, or death.

**FIGURE 3:**
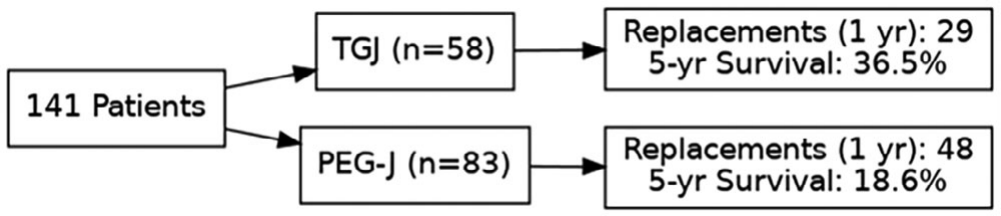
Flow diagram describing patient distribution and outcomes.

Regarding time to first replacement, treating death as a competing risk factor, Table 2 shows no significant difference in cumulative incidence of replacement within 1 year for TGJs and PEG‐Js (*p* = 0.389). In the first year, there were 29 TGJ replacements and 48 PEG‐J replacements. The association of tube type with overall survival was also not significant (*p* = 0.65). At 5 years, the survival was 36.5% and 18.6% in TGJ and PEG‐J, respectively (Figure 3).  A patient with a TGJ relative to a patient with PEG‐J had an increased risk of death of approximately 14% (HR = 1.135, 95% confidence interval [CI] 0.65, 1.97).  In a multivariable model including age and sex, PEG‐J patients had a decreased risk of death of approximately 2% (HR = 0.982, 95% CI 0.55, 1.74).

**TABLE 2:** Cumulative incidence of time to first replacement in PEG‐J and TGJ. P‐value 0.389.

Years	Cumulative incidence of 1st replacement
**PEG‐J**	
0.25	39.36 (30.05, 51.57)
0.5	49.27 (39.51, 61.46)
0.75	51.75 (41.94, 63.86)
1.0	59.19 (49.39, 70.92)
**TGJ**	
0.25	34.16 (23.70, 49.24)
0.5	50.81 (39.17, 65.90)
0.75	52.66 (40.98, 67.66)
1.0	52.66 (40.98, 67.66)

## 4 Discussion

This study aims to compare the outcomes of initial placement of TGJs placed via the Introducer technique to endoscopically placed PEG‐Js, which have not been previously described in the literature. In fact, the latest ASGE guidelines on feeding tubes do not discuss TGJs [4]. Thus, this study on outcomes of various initial percutaneous transgastric jejunal tubes is of paramount value to clinicians and the body of literature.

We postulated that the TGJ tube, with its larger overall diameter and sturdier build, would be less prone to flipping or clogging. However, our data showed that despite the increasing use of TGJ placement via the Introducer technique, there was no significant difference in the time to first replacement or the complications of tube clogging, flipping, or dislodgement compared to PEG‐Js, though there was a numerical tendency towards more of these complications in the PEG‐J group. This suggests that both tubes may be appropriate options for long‐term enteral nutrition. This finding also alludes to a likely limitation of our study, which is the fact that the proceduralist decides on the appropriate tube placement based on patient characteristics, including anatomy, availability of adequate sites for t‐fasteners, and provider experience. By choosing which procedure to perform based on clinical judgement, the longevity of a particular tube type and its complication rate could be skewed to favor a positive outcome. Our data shows that Introducer technique TGJ tubes were placed less often in patients with gastro‐jejunal obstruction or Billroth anatomy. A possible reason for this is the requirement for multiple sites on the anterior gastric wall for gastropexy, and this is less likely to be feasible when gastric resection has been performed. On the other hand, the Introducer technique TGJ tubes were placed more often for esophageal diseases, likely to overcome the vagal dysfunction that often occurs as a result of esophageal surgery. However, it is also important to consider that the selection of a particular feeding tube may relate to a proceduralist's comfort level with the placement of TGJ versus PEG‐J. Additionally, given that the underlying diseases in each patient group were different, there may be some selection bias accounting for the different types of tube placements in each group. Regarding anesthesia use at the time procedure, the type of anesthesia administered is at the anesthesiologist's discretion and does not have any influence on tube selection.

To further expand upon complications, it was initially hypothesized that PEG‐Js may have an increased incidence of tube clogging, flipping, or dislodgement [5]. This may be due to their two‐part system and smaller jejunal extension tube size [6, 7]. Thus, it was surprising that there was no significant difference between the groups, given that TGJs theoretically eliminate these structural deficits. There was a numerical tendency towards more clogging and flipping in the PEG‐J tubes, and as larger cohorts are identified in the future, this may become more significant. This potentially higher unplanned exchange rate may result in increased healthcare costs and utilization, suggesting that TGJs may be a more cost‐effective alternative. It is also important to consider that the various techniques used, introducer versus pull technique, for tube placement may also have played a role in the relative complication rates for each group. Further studies are needed to evaluate how the different techniques of placement may impact complications.

Regarding the relative risk of death in patients with PEG‐J versus TGJ, our multivariable model could only account for age and sex. This is due to the small cohort in our study, and for this reason, we unfortunately could not stratify the analysis based on indication for placement. In accounting for age and sex, though, our findings were also not statistically significant, despite the numerically increased percentage in risk of death for TGJ patients.

This study also did not evaluate several important factors, such as long‐term functional maintenance of each tube, ease of use, device cost, cosmetic outcomes, and quality of life. These factors are essential in determining the overall benefits of each tube. Further studies investigating these components would be invaluable.

Another limitation to discuss would be that patients were not followed beyond the initial date of replacement. Due to this, other complications and overall survival of TGJ versus PEG‐J were not captured. Additionally, given that some patients had died due to other causes prior to the tube's first expected replacement date, it is unknown how their survival may have impacted our data set. More studies are needed to determine the long‐term outcomes of patients receiving nutrition via TGJ versus PEG‐J, as well as the cost‐effectiveness of each relative tube

## 5 Conclusion

In conclusion, our study demonstrates that both initial TGJs and PEG‐Js are reasonable and safe options for long‐term jejunal feeding. Moreover, we demonstrate the safety and efficacy of initial jejunal feeding tube placement with TGJ rather than the previous practice of replacing PEG with TGJ after tract healing. The rising use of TGJ using the Introducer technique also requires further investigation regarding outcomes compared to other methods of TGJ placement, including interventional radiologic placement. Additionally, given that the procedure of choice is procedure‐dependent, it may be considered for more objective measures to be developed in the future to characterize which type of tube may be better suited for each patient, thus hopefully improving outcomes.

## Ethics Statement

**This research was approved by the Institutional Review Board**: IBR# 24–008867.

## Consent

N/A

## Conflicts of Interest

The authors declare no conflicts of interest.

## Clinical Trial Registration

N/A
